# Acute Ischemic Stroke during Extracorporeal Membrane Oxygenation (ECMO): A Narrative Review of the Literature

**DOI:** 10.3390/jcm13196014

**Published:** 2024-10-09

**Authors:** Konstantinos Themas, Marios Zisis, Christos Kourek, Giorgos Konstantinou, Lucio D’Anna, Panagiotis Papanagiotou, George Ntaios, Stavros Dimopoulos, Eleni Korompoki

**Affiliations:** 1 Medical School, National and Kapodistrian University of Athens, 157 72 Athens, Greece; kostasthemas@gmail.com (K.T.); mzisis01@gmail.com (M.Z.); 2 Department of Cardiology, 417 Army Share Fund Hospital of Athens (NIMTS), 115 21 Athens, Greece; chris.kourek.92@gmail.com; 3 Clinical Ergospirometry, Exercise & Rehabilitation Laboratory, 1st Critical Care Medicine Department, Evangelismos Hospital, National and Kapodistrian University of Athens, 157 72 Athens, Greece; stdimop@gmail.com; 4 Cardiac Surgery Intensive Care Unit, Onassis Cardiac Surgery Center, 117 45 Athens, Greece; konstantinougiorgosmd@gmail.com; 5 Division of Brain Sciences, Imperial College London, London SW7 2AZ, UK; l.danna@imperial.ac.uk; 6 First Department of Radiology, School of Medicine, National & Kapodistrian University of Athens, Areteion Hospital, 115 28 Athens, Greece; papanagiotou@me.com; 7 Department of Diagnostic and Interventional Neuroradiology, Hospital Bremen-Mitte/Bremen-Ost, 28205 Bremen, Germany; 8 Department of Internal Medicine, Faculty of Medicine, School of Health Sciences, University of Thessaly, 413 34 Larissa, Greece; gntaios@med.uth.gr; 9 Department of Clinical Therapeutics, National and Kapodistrian University of Athens, 157 72 Athens, Greece

**Keywords:** ischemic stroke, extracorporeal membrane oxygenation (ECMO), diagnosis, treatment

## Abstract

Ischemic stroke (IS) is a severe complication and leading cause of mortality in patients under extracorporeal membrane oxygenation (ECMO). The aim of our narrative review is to summarize the existing evidence and provide a deep examination of the diagnosis and treatment of acute ischemic stroke patients undergoing ECMO support. The incidence rate of ISs is estimated to be between 1 and 8%, while the mortality rate ranges from 44 to 76%, depending on several factors, including ECMO type, duration of support and patient characteristics. Several mechanisms leading to ISs during ECMO have been identified, with thromboembolic events and cerebral hypoperfusion being the most common causes. However, considering that most of the ECMO patients are severely ill or under sedation, stroke symptoms are often underdiagnosed. Multimodal monitoring and daily clinical assessment could be useful preventive techniques. Early recognition of neurological deficits is of paramount importance for prompt therapeutic interventions. All ECMO patients with suspected strokes should immediately receive brain computed tomography (CT) and CT angiography (CTA) for the identification of large vessel occlusion (LVO) and assessment of collateral blood flow. CT perfusion (CTP) can further assist in the detection of viable tissue (penumbra), especially in cases of strokes of unknown onset. Catheter angiography is required to confirm LVO detected on CTA. Intravenous thrombolytic therapy is usually contraindicated in ECMO as most patients are on active anticoagulation treatment. Therefore, mechanical thrombectomy is the preferred treatment option in cases where there is evidence of LVO. The choice of the arterial vascular access used to perform mechanical thrombectomy should be discussed between interventional radiologists and an ECMO team. Anticoagulation management during the acute phase of IS should be individualized after the thromboembolic risk has been carefully balanced against hemorrhagic risk. A multidisciplinary approach is essential for the optimal management of ISs in patients treated with ECMO.

## 1. Introduction

Extracorporeal membrane oxygenation (ECMO) is a temporary life support measure used in patients experiencing severe cardiac or respiratory failure that is refractory to optimized therapeutic interventions. There are two main types of ECMO support: Veno-arterial ECMO (VA-ECMO) and Veno-venous ECMO (VV-ECMO). VA-ECMO provides both cardiac and respiratory support and is primarily used in patients experiencing refractory cardiogenic shock or refractory cardiac arrest (e-CPR) [1]. However, Veno-venous ECMO (VV-ECMO) is indicated for respiratory support in patients experiencing acute respiratory failure refractory to conventional treatment [2]. Although recent randomized controlled trials have not shown a significant reduction in mortality rates for these patients [3,4,5], ECMO support remains a rescue treatment strategy for physicians when conventional treatment fails to maintain oxygen delivery to peripheral organs. The decision regarding the correct timing for ECMO or other mechanical circulatory devices (MCSs), such as Impella implantation for cardiogenic shock, remains crucial for the clinical outcome, especially in the context of complex clinical scenarios [6,7,8,9], and should be made thoughtfully and be based on predefined clinical, biochemical, echocardiographic and hemodynamic criteria [10].

ECMO circuit consists of a centrifugal pump, which provides a continuous flow, and a membrane oxygenator, which allows for oxygenation and decarboxylation of the blood. In VA-ECMO, the circuit includes an inflow cannula, which drains blood from the venous system (vena cava), and an outflow cannula, which delivers oxygenated blood back into the arterial system through femoral or axillary access with appropriate blood pump flow according to tissue perfusion. The timing and selection of patients for left ventricle (LV) unloading in VA-ECMO patients are of paramount importance to avoid pulmonary edema and LV thrombosis and, as a result, improve clinical outcomes [11,12].

In most cases, the addition of an intra-aortic balloon pump (IABP) is necessary to improve coronary perfusion as a first line LV unloading approach, but other LV supportive measures, such as Impella, Tandem Heart and ProtekDuo, can also be helpful in thoroughly selected patients. The selection of patients is based mainly on individual echocardiographic and hemodynamic assessment [11,13]. The decision to unload LV without increasing the risk of complications due to an additional mechanical circulatory support (MCS) device is complex and requires a multidisciplinary team approach. Bleeding, coagulation disorders, vascular complications (limb ischemia, thromboembolism) and hemolysis are the main complications that might occur with the addition of MCS [11,13].

In VV-ECMO, the outflow cannula facilitates the delivery of oxygenated and decarboxylated blood to the right atrium, ensuring normal blood gas exchange through femoral or jugular access. The use of VV-ECMO has significantly expanded in recent years, mainly due to critically ill COVID-19 patients [14].

ECMO support confers favorable outcomes in select critically ill patients and life-threatening situations, but it remains a complex high-risk intervention associated with several complications, including cerebrovascular events that affect patients` outcomes and quality of life. ECMO use has been mainly associated with thrombotic complications; hence, anticoagulation therapy should always be started in patients receiving ECMO support [15]. Nevertheless, despite careful administration of anticoagulation therapy, there is always a significant risk of bleeding events. Thus, the risk of thromboembolism, which includes the risk of an acute ischemic stroke, should be carefully balanced against the risk of bleeding, which includes the risk of an intracranial hemorrhage [16,17,18]. The decision regarding the type of ECMO needed has been associated with the occurrence of specific cerebrovascular complications: VA-ECMO is mainly associated with ISs [19], whereas VV-ECMO is associated with hemorrhagic strokes [20]. Early recognition of cerebrovascular events is of paramount importance for immediate treatment in ECMO patients.

Although a stroke is a severe complication and a leading cause of mortality in ECMO patients, there are limited data on the incidence, risk factors and management of ischemic strokes during ECMO. The aim of this review is to summarize the existing evidence and provide more insights into the diagnosis and treatment of ischemic strokes in patients under ECMO support.

## 2. Epidemiology of Cerebrovascular Complications in ECMO Patients

Acute central nervous system (CNS) complications associated with the use of ECMO include ischemic stroke, intracranial hemorrhage (ICrH), hypoxic ischemic brain injury (HIBI), subarachnoid hemorrhage (SAH) and seizures. Some studies also report brain death in the group of CNS complications. Overall, the incidence of such complications ranges from 4.5% to 16% across different studies, while the mortality rate has been reported to be as high as 50–89%, as presented in Table 1.

An ischemic stroke is a common complication in patients supported with ECMO. Across observational studies, the incidence rate ranges between 1 and 8%, and the mortality rate associated with ischemic strokes is between 44 and 76% [17,28]. Ischemic strokes are more common in male patients compared to females. The median age of patients experiencing ISs under ECMO support has been reported to range from 50 to 62 years.

Of note, the true incidence rate of CNS complications, and especially of acute IS, may be higher than reported in observational studies. Indeed, several postmortem reports showed that the number of cerebral infractions was underreported [29]. The significant number of clinically unreported infarctions may be explained by the lack of comprehensive clinical assessment, which is further confounded by the severity of the critical illnesses of the patients and the use of sedation. Furthermore, performing neuroimaging in these patients can be logistically challenging, as they are at especially high risk of being transferred to the radiology suite.

With regard to the type of support, neurological complications are more common with VA-ECMO than with VV-ECMO. This might be explained by the use of VA-ECMO in extracorporeal CPR (E-CPR). There is no difference in the incidence of total neurological complications between VA and VV-ECMO when excluding patients that received extracorporeal CPR [21]. However, the occurrence of ischemic strokes during the use of VA-ECMO remained significantly more common. The same was also observed in COVID-19 patients [30]. COVID-19 patients under ECMO support had a higher incidence of CNS complications and higher mortality rates than other patient groups [31]. However, the rates of ischemic strokes in patients supported by ECMO due to COVID-19 did not differ significantly from other patients’ categories, with an incidence rate for acute ISs of between 2 and 6% [30,31,32,33].

Ischemic strokes in pediatric ECMO patients are uncommon, although the impact on the developing brain can be severe, with the potential for lifelong neurologic injury [34]. The overall prevalence of strokes in pediatric ECMO patients has been reported at between 3 and 6% [35]. Indeed, diagnoses of stroke were shown to be significantly higher in older children (1–18 years) than in infants (<1 year), but there was a significantly higher prevalence of strokes in pediatric ECMO patients with congenital heart disease, mostly beyond the first year of life [34].

## 3. Pathophysiology and Risk Factors

Several mechanisms of ischemic strokes during ECMO have been identified, the majority of which are thromboembolic events and cerebral hypoperfusion [28,36,37]. This is of particular importance because strokes of thromboembolic origin are accompanied by a higher mortality rate and lead to worse functional outcomes [38]. With regard to the origin of infarcts, small focal ischemic lesions have been associated with air or thrombotic microemboli, while larger lesions are associated with larger thrombotic emboli [36,37,39]. The mechanisms of thrombus formation implicate both special characteristics of the ECMO circuit and the underlying prothrombotic condition of patient. More specifically, the interface between the blood components and the ECMO circuit composite material surface can activate the coagulation cascade, while patients’ comorbidities and critical illnesses predispose patients to both thrombotic and bleeding complications [40]. The pathophysiology of cerebral hypoperfusion in patients undergoing ECMO treatment involves loss of cerebral autoregulation, i.e., cerebral vasoconstriction, as a result of underlying hemodynamic instability [37,41]. Other proposed possible mechanisms include the direct connection of an ECMO circuit with the arterial network, bypassing the lungs and allowing embolic material direct access to the cerebral vessels [26]. Furthermore, VA-ECMO provides laminar blood flow which, in contrast to natural pulsatile flow, may lead to endothelial dysfunction and impairment of the cerebral vascular autoregulation mechanisms [28]. Another proposed mechanism is differential hypoxia, a situation where native cardiac output provides poorly oxygenated blood—blood originating from dysfunctional lungs (atelectasis, lung infection, pneumothorax, ARDS etc.)— to the brain, causing impairment of brain tissue oxygenation [37,42].

Several studies have reported risk factors predicting the occurrence of an ischemic stroke. The usage of VA-ECMO is linked with an increase in the incidence rate of ISs [21,28]. A high platelet count number (>350 × 109/L) and central site, over peripheral, cannulation in VA-ECMO predispose to higher rates of acute ISs [19]. However, a recent study has found no significant difference between cannulation sites [43]. Lower pre-ECMO acidosis and higher concentrations of lactic acid (>10 mmol/L) were independently associated with acute ischemic strokes, reflecting a patient’s refractory respiratory distress or hemodynamic instability status prior to the use of ECMO [17,30,44]. Higher PaO_2_ levels on the first day of ECMO support were associated with acute IS occurrence [26,27]. It is also known that early hyperoxia is related to poor neurologic outcomes in ECMO patients experiencing ischemic strokes [45]. A possible explanation is increased oxidative stress leading to reperfusion injury [46]. A higher fast reduction in PaCO_2_ levels between ECMO initiation and the next 24 h was also found to be an independent risk factor [27]. This is in accordance with other studies [47] and the established physiological mechanism, where lower PaCO_2_ levels (hypocapnia) led to cerebral vasoconstriction and thus reduced cerebral blood flow [48].

Finally, impairment of coagulation is not uncommon in ECMO patients, leading to microthrombi formation, coagulation dysregulation and thromboembolic events. Disseminated intravascular coagulation (DIC), hemolysis and gastrointestinal hemorrhaging have been associated with acute ISs [17].

A summary of all independent risk factors regarding acute ischemic strokes is presented in Table 2.

## 4. Diagnostic Algorithms and Neurological Monitoring

Early recognition of neurological deficits is crucial for prompt therapeutic interventions in ECMO patients experiencing ischemic strokes. Multimodal neurologic monitoring (MNM) and daily clinical assessment could be useful strategies for the early identification of neurologic deficits and deterioration, even in severely ill patients under sedation [49].

Any ECMO patient with suspected stroke should receive brain computed tomography (CT) and CT angiography (CTA) of the neck vessel, as well as circle of Willis for the identification of large vessel occlusion (LVO) and the assessment of collateral blood flow, according to international guidelines [50,51]. CT perfusion (CTP) can further guide the detection of viable tissue (penumbra), especially in the case of strokes of undetermined onset time. However, it should be stressed that brain imaging with CT, CTA and CTP for I wothe initial assessment of acute ischemic strokes in ECMO patients may be very challenging. This is because cerebral blood flow and hemodynamics are significantly impaired in these patients because of high-pressure arterial inflow in the setting of VA-ECMO, resulting in artifacts often being interpreted as false positive findings of LVO and perfusion deficits on CTA and CTP, respectively [52,53]. These false positive results mimicking large vessel occlusion with irreversibly infarcted tissue can be attributed to cannulation through the axillary artery high-pressure non-opacified blood flow competing with the systemic contrast-opacified blood, causing unilateral non-opacification of the extracranial and intracranial vessels [52]. These changes are visualized as perfusion asymmetry in CTP, influencing the accuracy of conventional brain imaging interpretation. Close collaboration between an ECMO team and neuroradiologists, by incorporating expected hemodynamic changes produced by ECMO, flow rate in the ECMO system and the degree of residual left ventricular function, could facilitate the interpretation of CTA imaging [53]. In a case where an ischemic stroke, with positive CTA imaging for LVO, is suspected, catheter angiography (digital subtraction angiography (DSA)) may provide an accurate and reliable diagnostic technique to exclude or confirm vessel occlusion [52], with the opportunity to, at the same time, proceed with mechanical thrombectomy.

Although magnetic resonance imaging (MRI) provides the modality of choice in uncertain cases or in cases of suspected brainstem infarct, its use is challenging in cases of ECMO support. Established neuroimaging protocols ideally requiring restricted time in the radiology suite are essential for the assessment of ECMO patients [54]. For a thorough assessment of both ischemic and hemorrhagic lesions, MRI protocols should include the multiplanar sequences consisting of T1- and T2-weighted, fluid-attenuated inversion recovery (FLAIR), diffusion-weighted imaging (DWI) and susceptibility-weighted images (SWI) [55]. However, the transfer of these patients outside of the intensive care unit (ICU) poses significant risks. The SAFE MRI-ECMO study showed that a low-field portable MRI is a safe and logistically feasible option to use with ECMO patients for identifying acute infarcts before their visualization in CT scans [54,56]. On the other hand, a significant challenge to MRI accessibility is that the ECMO circuit itself can be incompatible with the MRI magnet [37].

Alternative, flow-based imaging modalities, such as the transcranial doppler (TCD) and carotid Doppler ultrasonography, can also be considered in these patients [57]. TCD is a very useful tool in treating patients on ECMO as it provides a non-invasive bedside technique that facilitates indirect blood flow monitoring by the estimation of mean flow velocities (MFV) and pulsatility indices (PI), allowing for comparison between the two middle cerebral arteries [58]. It provides direct measurements of the pattern of cerebral blood flow, hemodynamic reserve and microembolic signals [57]. TCD may also assist with the estimation of hemorrhagic risk. An increased MFV with low PI has been associated with an enhanced risk of intracerebral hemorrhage, similarly to cerebral hyperperfusion syndrome [59]. TCD has been used in both pediatric and adult ECMO populations [60].

Another useful non-invasive bedside modality for cerebral hemodynamic monitoring could be cerebral near infrared spectroscopy (NIRS) [61]. It has been evaluated in ECMO patients for its potential to detect brain injury in patients on both VA and VV-ECMO [62]. The neurological pupil index (NPi) is an automated pupillary assessment tool that assesses minimal and maximal pupil sizes, constriction velocity and latency, which can be used as an early, non-invasive indicator of increasing intracranial pressure (ICP) [63] and could even predict 90-day mortality [64]. NPi has some disadvantages, as it can be affected by ambient light, sedation analgesia and high concentrations of opioids [65].

**Figure 1:**
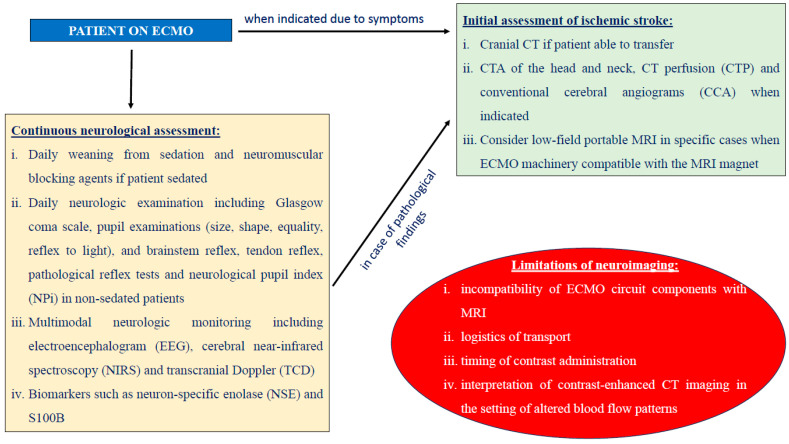
Proposed algorithm for early ischemic stroke recognition in patients under ECMO support.

Finally, biomarkers, such as neuron-specific enolase (NSE) and S100B, have been proposed as valuable prognostic tools and present an association with 28-day mortality and CT findings [66]. Biomarkers could be used along with MNM to identify patients who are at a higher probability of worse outcomes. It should be implemented in every patient at increased risk on ECMO, and it includes daily neurologic examinations of patients without sedation, portable head CT, electroencephalogram (EEG) and transcranial doppler. It has been also proven to be safe and feasible for these patients [49]. A proposed algorithm suggests that patients undergo continuous video EEG monitoring for the first 24 h, daily transcranial doppler for the first three days and head CT on days one and three after ECMO initiation. These diagnostic techniques could be repeated subsequently on an individual basis in cases where there is clinical indication. Neurologic examinations of patients on ECMO support include the Glasgow Coma Scale and pupil examinations (size, shape, equality, reflex to light), as well as brainstem reflex, tendon reflex and pathological reflex tests. The use of MNM does not only facilitate prevention, but also plays a role in prognosis and clinical decision-making in patients on ECMO support [49]. In the context of an acute IS, the National Institute Health Stroke Scale (NIHSS) should be used for the assessment of the neurological deficit. A proposed algorithm for IS recognition in patients under ECMO support is proposed in Figure 1, based on international stroke guidelines [50] and neurological monitoring consensus guidelines for ECMO patients [67].

## 5. Management

In the acute phase of an ischemic stroke, the main goal of all management strategies is to rescue still-viable brain tissue surrounding the necrotic area (penumbra) using thrombolysis and mechanical thrombectomy, avoid and treat acute complications and prevent stroke recurrence [50]. However, in most ECMO-supported patients, the use of necessary anticoagulation therapy prior to the ischemic event is a contraindication for intravenous thrombolysis [68]. Thrombolysis after the reversal of anticoagulation activity with prothrombin complex concentrates or specific reversal agents is challenging in ECMO patients because of the very high risk of thromboembolism and ECMO circuit thrombosis. Although there are some data on thrombolysis in selected patients experiencing ISs and receiving factor Xa inhibitors after the measurement of anti-Xa activity [68], data on ECMO patients experiencing ISs and receiving anticoagulation therapy are lacking. Thrombolysis with rtPA administered for life-threatening oxygenator thrombosis has been given effectively in ECMO patients but at lower doses than in thrombolysis for ischemic strokes (i.e., 5–20 mg) [69]. Consequently, mechanical thrombectomy is the preferred therapeutic option in eligible patients [70,71]; hence, proven LVO in CTA is essential for endovascular treatment. The choice of arterial vascular access to perform MT should be discussed between interventional radiologists and ECMO specialists, with the femoral artery opposite to arterial inflow cannula providing a feasible option in most cases. Stroke severity assessment is often challenging in ECMO patients who are critically ill or sedated; therefore, MNM and daily neurological examination would be helpful in estimating any change in the neurological status.

The use of anticoagulation is necessary in most ECMO cases for circuit clotting prevention. The latest ELSO guidelines suggest the use of unfractionated heparin (UFH) or direct thrombin inhibitors (bivalirudin or argatroban) in cases of heparin-induced thrombocytopenia syndrome, with the choice being made by the clinicians on a case-by-case basis [72]. In multiple studies, the main anticoagulant used was UFH, monitored using activated partial thromboplastin time (aPTT) or activated coagulation time (ACT), with fewer cases using bivalirudin and various other agents [25,32,33,44].

The choice and timing of the initiation or resumption of antithrombotic therapy during a thromboembolic ischemic stroke is a complex task, as the balancing between thrombotic and hemorrhagic risk requires a holistic assessment and multidisciplinary approach [73]. For patients at especially high hemorrhagic and thrombotic risk, it has been suggested that the early cessation and careful resumption of anticoagulation is feasible 1–2 days after the event, assuming a stable neurological situation and CT imaging hemorrhagic transformation [25]. The anticoagulant of choice is UFH with the aPTT goal being 50–70 s.

The combination of bleeding and thrombosis suggests either heparin-induced thrombocytopenia/thrombosis (HIT) or disseminated intravascular coagulation [74]. The treatment of HIT, in the presence of positive antibodies or strong clinical suspicion, may include a switch from heparin to either bivalirudin or argatroban [75]. Patients with an elevated aPTT, elevated prothrombin time, low fibrinogen, elevated D-dimer or increased fibrinolysis should be further investigated for disseminated intravascular coagulation [76]. Nevertheless, in the case of a failure of the membrane oxygenator, the ECMO circuit should be replaced if a thrombus is causing disseminated intravascular coagulation [75]. The daily assessment of patients on ECMO should include a platelet count, PT/international normalized ratio (INR) and aPTT is important, with a goal of a platelet count above 50,000 and the correction of PT/INR or fibrinogen levels, if there is clinical evidence of bleeding [75].

**Figure 2:**
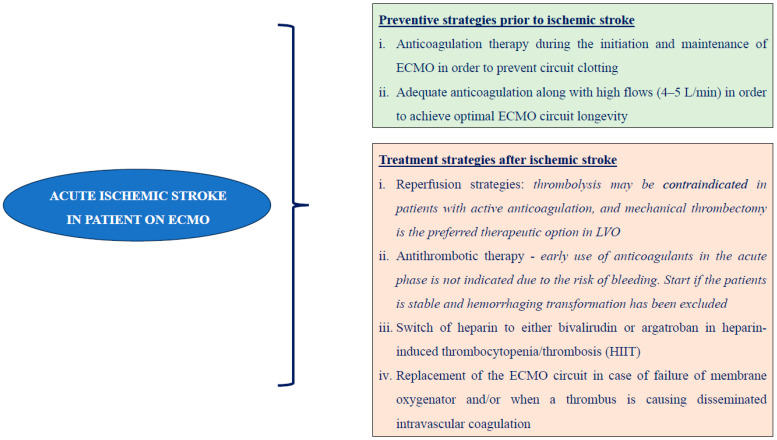
Proposed treatment algorithm for ischemic strokes and other complications in patients under ECMO support.

Viscoelastic Point of Care (POC) monitoring also demonstrates potential benefits for coagulation management in ECMO patients that might have a role in clinical decision-making [77]. A proposed treatment algorithm for patients on ECMO with acute ISs and other complications is demonstrated in Figure 2, which is based on international guidelines [50,51,67].

## 6. Conclusions

An acute ischemic stroke is a common complication among ECMO-supported patients, accompanied with high mortality rates. A continuous neurological assessment combined with a prompt usage of neuroimaging techniques may increase early stroke detection and recognition. Ischemic stroke management in ECMO patients is a highly complex task, requiring a multidisciplinary team approach with careful balancing of anticoagulation against the hemorrhagic risk of these patients. While thrombolysis is usually contraindicated due to high bleeding risk, mechanical thrombectomy likely represents the treatment strategy of choice in thoroughly selected patients. Proposed algorithms need to be validated using a dataset of ECMO patients to demonstrate the benefits on the outcomes. Further studies are required to provide evidence regarding the optimal diagnostic and therapeutic approach.

## Figures and Tables

**Table 1:** Incidence and mortality rate of CNS complications and ischemic strokes during ECMO.

Author	Study Design	Number of Patients	Incidence of CNS Complications (%)	Mortality of CNS Complications (%)	Incidence of Ischemic Stroke (%)	Mortality of Ischemic Stroke (%)
Nasr and Rabinstein [18]	Retrospective	23.950	7.7	49.9	4.1	44.3
Le Guennec et al. [19]	Retrospective	878 (all VA ECMO)	7.4	N/A	5.3	57
Shoskes et al. [21]	Systematic review and meta-analysis	16,063 (VA ECMO: 8221 VV ECMO: 7842)	16	N/A	7	N/A
Sutter et al. [22]	Systematic review and meta-analysis	N/A	13	83	5	84
Chapman et al. [16]	Retrospective cohort study	412	13.3	65	7	N/A
Lorusso et al. [23]	Retrospective	4522 (all VA ECMO)	15.1	89	3.6	74
Lorusso et al. [24]	Retrospective	4998 (all VV ECMO)	7.1	75.8	1.7	68.2
Prokupets et al. [25]	Retrospective	156 (all VA ECMO)	12.4	78.9	8.4	85
Cho et al. [17]	Retrospective	15,872 (all VV ECMO)	5.1	74–77	1.4	68
Cho et al. [26]	Retrospective	10,342 (all VA ECMO)	7.9	N/A	3.9	76
Hwang et al. [27]	Retrospective	20,297 (all VA ECMO)	4.5	N/A	3	65

**Table 2:** Risk factors predicting ischemic strokes during ECMO.

Author	Study Design	No of Patients	Independent Risk Factors for AIS	OR (95% CL)	*p* Value
Iacobelli et al. [28]	Retrospective single center cohort study	275	Use of VA ECMO	4.86 (1.8–13.12)	0.002
Le Guennec et al. [19]	Retrospective	878 (all VA ECMO)	Use of central VA ECMO	3.2 (1.5–6.6)	0.002
			PLTs > 350 10^9^/L	3.8 (1.4–10.7)	0.01
Cho et al. [17]	Retrospective	15,872 (all VV ECMO)	pre ECMO pH	0.10 (0.03–0.35)	<0.001
			DIC	3.61 (1.51–8.66)	0.004
			Hemolysis	2.27 (1.22–4.24)	0.010
			GI Hemorrhage	2.01 (1.12–3.59)	0.019
Cho et al. [26]	Retrospective	10,342 (all VA ECMO)	pre ECMO pH	0.21 (0.09–0.49)	<0.001
			higher PO_2_ (10 mm Hg) at 24 h	1.01 (1–1.02)	0.009
			Renal Replacement Therapy (RRT)	1.49 (1.14–1.94)	0.004
Omar et al. [44]	Retrospective chart review	171	pre-ECMO lactic acid > 10 mmol/L	7.586 (1.396–41.223)	0.019
Shoskes et al. [21]	Meta Analysis	16,063 (VA ECMO: 8221/VV ECMO: 7842)	Use of VA ECMO	N/A	0.001
Hwang et al. [27]	Retrospective	20,297 (all VA ECMO)	lower ΔPaCO_2_ (10 mmHg) at 24 h	0.990 (0.984–0.996)	0.0009
			higher PO_2_ (10 mm Hg) at 24 h	1.002 (1.001–1.002)	0.0006

## Data Availability

Not applicable.
